# Basal Cell Adenoma of Palate, a Rare Occurrence with Review of Literature

**Published:** 2015-09

**Authors:** Achla Bharti Yadav, Anjali Narwal, Anju Devi, Sanjay Kumar, Sumit Kumar Yadav

**Affiliations:** a Dept. of Oral Pathology, Postgraduate Institute of Dental Sciences, Pt. B. D Sharma University of Health Sciences, Rohtak, India.; b Dept. of General Pathology, Post Graduate Institute of Medical Sciences, Pt. B. D Sharma University of Health Sciences, Rohtak, India.; c Dept. of Orthodontics & Dentofacial Orthopedics, Mithila Minority Dental College & Hospital, Darbhanga, Bihar, India.

**Keywords:** Basal cell adenoma, Basaloid, Immunohistochemistry, Isomorphic

## Abstract

Basal cell adenoma is an uncommon benign epithelial neoplasm of salivary gland which derives its name from the basaloid appearance of tumor cells and accounting for 1-2 % of all salivary gland epithelial tumors. This tumor usually arises in the major salivary glands, with the parotid being the most frequent site of occurrence, followed by the upper lip; while it is very rare in the minor salivary glands. Microscopically, it is composed of isomorphic cells similar to basal cells with nuclear palisading. We report a case of BCA presenting as an asymptomatic swelling over the right side of palate of 55-year-old female patient. A follow-up of 1 year revealed no recurrence. This report emphasizes the rare site of occurrence of this tumor and briefly reviews the literature.

## Introduction

World Health Organization defined basal cell adenoma (BCA) as an idiosyncratic benign neoplasm composed of monomorphic population of basaloid epithelial cells, organized with a prominent basal cell layer and a distinct basement membrane like material; however, the myxochondroid stromal component characteristic of mixed tumor is absent.(1-2)

Kleinsasser and Klein (1967) first used the term basal cell adenoma to describe an encapsulated, slow growing, merely epithelial neoplasm composed of discernible basal cells arranged in the form of solid sheets or nests and trabecular/ tubular cord like pattern.(3) The most common site of occurrence is the parotid gland(4-5)followed by upper lip with a decreasing incidence in palate, buccal mucosa and lower lip.(4) It usually occurs in patients over 50 years of age with slight female predilection.(6) Concerning its Clinical presentation, it exhibits as a slow growing, asymptomatic, movable, round or oval, normal-colored sub mucosal mass measuring less than 3 cm in diameter.(4)

Microscopically, BCAs are well circumscribed and encapsulated by fibrous connective tissue.(1) Tumormass consists of proliferation of terminal duct epithelial cells forming is lands or sheets supported by a sparse fibrous stroma, and presence of a small number of myoepithelial cells.(4, 2) It has distinct basement membrane and often exhibit palisading of basal layer cells. They also lack the myxochondroid areas characteristic of pleomorphic adenoma.(7) Histopathologically, BCA can be divided into four subtypes, i.e. solid, trabecular, tubular, and membranous.(2)

Basal cell adenoma is an uncommon tumor with palate being the rarest site for its occurrence.(4) To the best of our knowledge, only five cases involving the palatal minor salivary glands have been reportedin the English literature till date. The present case is most likely the sixth case(4, 14-17) which highlights the rarity of this tumor with regard to its site of origin/ occurence.

## Case Report

A 55-year-old female reported to the department of Oral and Maxillofacial Surgery with a chief complaint of painless swelling on the right side of palate since two months. General clinical examination was unremarkable with no history of weight loss, diabetes, hypertension, tuberculosis, or some other systemic diseases.

**Figure 1:**
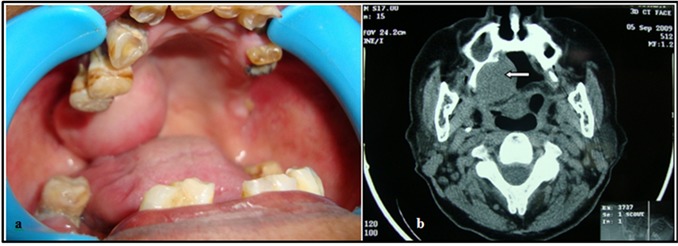
a: Intraoral view shows swelling on the right hard palate  b: CT scan demonstrates soft tissue density mass on the right side of palate (arrow)

Intraoral examination showed a localized asymptomatic swelling over right side of palate. When inspected, it was ovoid in shape, measuring 2.5×2cm with well-circumscribed borders. Palatal swelling was extending anterio-posteriorly from canine to retrotuberosteal region and laterally from midpalate to bucco alveolar region (Figure 1a). On palpation, swelling was firm, non-tender, non-compressible, smooth-surfaced with freely mobile overlying mucosa. Clinical examination of head and neck did not reveal any palpable lymph nodes or masses. Radiographic examination with computerised tomographic (CT) scan revealed soft tissue density mass on right side of oral cavity, posteriorly hanging from the hard palate with scalloping of the overlying bone (Figure 1b).

**Figure 2:**
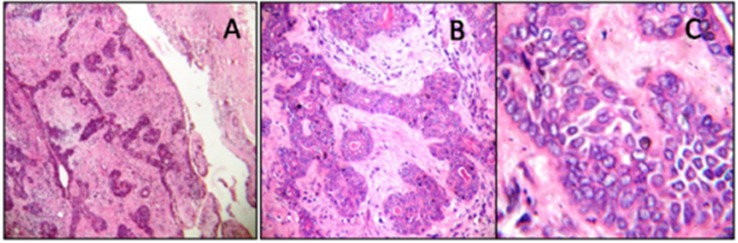
Histopathology of the basal cell adenoma (H & E stain) (A) Encapsulated tumormass arranged in the form of solid nests and trabeculae (4x). (B) Tumor islands composed of peripheral palisaded cuboidal to columnar shape cells with round hyperchromatic nuclei and relatively rounded central cells (10 X). (C) Monomorphic basaloid cells (40X).

Microscopic examination of incisional biopsy specimen showed principally basaloid cells arranged in clumps with hyperchromatic nuclei and increased nuclear-cytoplasmic ratio. A probable diagnosis of benign monomorphic adenoma of salivary gland was made. Surgery was planned and tumor was excised along with a safe margin after ligating greater palatine vascular bundle and the defect was repaired by buccal fat pad (BFP). The excised pathology was submitted to the department of Oral and Maxillofacial Pathology where the routine tissue processing was carried out. The histopathological examination of the excised specimen revealed well-encapsulated tumormass which was composed of monomorphic basaloid cells arranged in the form of solid nests and trabeculae pattern in the loose connective tissue stroma. The peripheral cuboidal cells were in palisading arrangement with round hyperchromatic nuclei and scanty cytoplasm. The central cells of the nests were larger with modest cytoplasm, indistinct cell borders and a pale staining oval nucleus having inconspicuous nucleoli. Few spindle shaped cells suggestive of myoepithelial cells were present in the stroma surrounding the basaloid tumor nests. Overall features were consistent with the diagnosis of BCA (Figure 2). Entire healing was uneventful and after a follow-up of 1 year, there were no signs of recurrence.

## Discussion

The basal cell adenoma was once considered to be a type of monomorphic adenoma.(4) Kleinsasser and Klein were the first to designate the term basal cell adenoma and to establish it as a distinct clinical and pathologic entity in 1967.(1-2,7) Gardner and Daley described the distinguishing features of basal cell adenoma and canalicular adenoma to document these as two separate entities.(3) In the revised WHO classification of salivary gland tumors (1991), basal cell adenomas were included in the benign epithelial neoplasm, excluding the word  monomorphic(1, 4) and define it as a tumor of isomorphic basaloid cells organized with a noticeable basal cell layer and separate basement membrane like structure and myxochondroid stromal component of mixed tumor was not present.(8) Gardner and Daley described the histologic subtypes of BCA which include solid, trabecular, tubular, and membranous,(2) with the solid variant being the most common.(9) Batsakis reported the first case of BCA in the American literature in 1972, and suggested that the intercalated duct or basal cell is the histogenetic source of BCA.(10)

Salivary gland tumors are uncommon and constitute 2-6.5% of all the head and neck neoplasms(4, 11) and tumors of minor salivary gland account for less than 25% of all salivary neoplasm.(4) Basal cell adenoma accounts for 1–2% of all salivary gland epithelial tumors.(1-2,7)

BCA arises almost exclusively in adults and the mean age of the patient is 57.7 years with the peak incidence in sixth decade of life;(1-2) however, unusual cases of congenital BCAs have also been reported.(12-13) Several studies support a slight predominance of this tumor in females(8, 11, 14-15) although membranous variant has an equal gender distribution.(1)

Palate is the most common site for minor salivary gland tumors with 40-80% of incidence.(15) But it is relatively an uncommon site for BCA(14) which usually arises in the major salivary glands with the parotid being the most frequent site of occurrence followed by minor salivary glands of upper lip.(9, 16) In the opinion of Fantasia and Neville, BCA typically occurs in older patients with mean age of61 years and most commonly involves the upper lip.(17) In 1991, 160 cases of BCA were registered at the Armed Forces Institute of Pathology (AFIP), which constitute 1.8% of all benign epithelial salivary gland tumors, out of those BCA 75% were reported in the parotid gland and 20% in the minor salivary glands of the upper lip.(2, 9) In our case the tumor presentation was in minor salivary glands of the palate which is considered as a rare site for BCA as only five cases have been reported in literature till date.(4, 14-16,18)

In our case, the patient was female in sixth decade of her life which fits into the age range and gender predominance of BCA.The same trend as of BCA, regarding age, gender and clinical presentation was seen in our case except the site of occurrence.

Biopsy is recognized as the most precise method for diagnosis of BCA, although some authors advocate FNAC if physical access to the tumor is available.(10, 19) BCAs are mostly well circumscribed and encapsulated by fibrous connective tissue. Histologically, it has four variants named as solid, tubular, trabecular, and membranous; solid being the most common, but each tumor has combination type.(1-2) These variable patterns of BCA consists of 2 types of cell populations (basaloid cell and luminal duct cells).(8) The first cell type is small cuboidal or columnar shaped, present peripherally in a palisading arrangement within the tumor nests or cords, with round deeply stained nuclei and little discernible cytoplasm. The second cell type, presenting centrally, is larger with modest cytoplasm, indistinct cell borders and a pale staining oval nucleus. Sharp demarcation is present between the neoplastic cells and the surrounding stroma.(1-2,20)

Histopathological examination has been accepted as a gold standard for confirmatory diagnosis of BCA. Although immunohistochemistry (IHC) examinations non-specific, variable, and somewhat dependent on histologic subtype of BCA,(1) however, it can be used as an adjunct in the differentiation of these tumors. Different investigators reported varying results of IHC staining in basal cell adenomas. Basaloid cells in trabecular and solid areas express cytokeratins but the number of reactive cells varies from few to many. Similarly, S-100 protein, smooth muscle actin (SMA), and vimentin show immunopositivity among the peripheral tumor cells of BCAs. Carcinoembryonic antigen (CEA) and epithelial membrane antigen (EMA) reactivity is mostly confined to luminal cells.(8) Selective positivity for pancytokeratin, S-100 and smooth muscle actin was reported by Hemachandran *et al. *which suggests the role of myoepithelial cells in histogenesis of BCA.(21)

In spite of distinctive appearance of BCA, other primary tumorswhich can simulate its basal cell features and causes difficulty in diagnosis are pleomorphic adenoma (PA), adenoid cystic carcinoma (ACC), basal cell adenocarcinoma (BCAC). Lack of myxo-chondroid stroma and parenchyma well demarcated by a distinctive basal membrane differentiate BCA from pleomorphic adenoma.(1-2) The most difficult differential consideration is adenoid cystic carcinoma. (2) There are two characteristics that assist in distinguishing these lesions. One is the circumscription of the basal cell adenoma which differs from invasive pattern of adenoid cystic carcinoma. The other is the absence of vascularity in the microcysticareas of adenoid cystic carcinoma, which differs from the numerous endothelial lined channels in basal cell adenoma.(7) Other distinctive features of BCA are lack of perineural invasion andincreased bland tumor cell population. Differentiation of BCA from BCAC is predominantly architectural, as the cellular composition of these tumors is similar. In contrast to BCA, BCAC is a low grade carcinoma which exhibit unencapsulated growth pattern and invades into the adjacent soft tissue, often associated with perineural or vascular invasion.(1)

Immunohistochemically, the outer tumor cells in pleomorphic adenoma shows a heterogeneous distribution of glial fibrillary acidic protein (GFAP) which shows negativity for tumor cells in BCA.(11) Although Williams *et al.* found rare faint-staining of GFAP in BCA, others have not seen this.(8) In comparison to AdCC, expression level of MMP9, lamin in and CD117 were lower in cribriform type of BCA (cBCA) as reported by Li BB *et al.*(22) IHC including Ki-67 labeling and beta-catenin should be performed to differentiate BCA from BCAC for a definitive diagnosis.(9) Ki67 staining of greater than 5% of the cells supports the diagnosis of BCAC.(10, 19)

The recurrence rate is 25-37% for the membranous variant of BCA,(9) possibly related to its multifocal nature, which impairs complete removal.(4) Although exceedingly rare, malignant transformation of BCA has been reported.(1) Therefore, it is necessary to perform complete tumor excision. This approach was used in our case and the postoperative period was uneventful with no signs of recurrence even after a follow up of 1 year.

## Conclusion

Herein, the goal of this paper is to add on to the literature one more case of this rare tumor of BCA arising from minor salivary glands of hard palate and to sensitize the dental surgeons to include BCA as a differential diagnosis of palatal swellings. 
